# Clinical and Pharmacologic Profile of a Subcutaneous Lecanemab Formulation

**DOI:** 10.1002/alz70859_104691

**Published:** 2025-12-25

**Authors:** Steven Hersch, Michelle Gee, Thomas Doherty, Natasha Penner, Larisa Reyderman, Anna Patten, Shobha Dhadda, Michael C. Irizarry, Lynn D Kramer

**Affiliations:** ^1^ Eisai Inc., Nutley, NJ USA; ^2^ Eisai Ltd., Hatfield United Kingdom; ^3^ Eisai Ltd., Hatfield, hertfordshire United Kingdom

## Abstract

**Background:**

Lecanemab 10 mg/kg every 2 weeks (Q2W) intravenously was shown in the Clarity AD trial to slow the progression of Alzheimer’s disease (AD). Pharmacokinetic/pharmacodynamic (PK/PD) modeling demonstrated that lecanemab average steady‐state concentrations (Cav, ss) are associated with amyloid plaque lowering and efficacy, while maximum steady‐state concentrations (Cmax, ss) are correlated with ARIA‐E. A subcutaneous lecanemab formulation was hypothesized to have similar or better safety profile, with lower rates of systemic administration reactions. Herein, we provide an update on the clinical results from the subcutaneous lecanemab development program.

**Methods:**

Clarity AD is a phase 3, double‐blind, placebo‐controlled study of 18‐month treatment duration with an ongoing open‐label extension (OLE) in patients with early AD. Eligible patients were randomized to placebo or 10 mg/kg lecanemab biweekly. Subcutaneous dosing is under development based on pharmacokinetic (PK) and pharmacodynamic (PD) modeling, bioavailability data, as well as clinically in a substudy of the OLE. A subset of participants in the subcutaneous substudy were lecanemab naïve. Validated anti‐drug antibodies (ADA) and neutralizing antibody (Nab) assays were performed using a tiered approach.

**Results:**

In the Clarity AD phase 3 study, lecanemab subcutaneous weekly dosing with a single 360 mg autoinjector has low rate of ADA (2%) with low titers and maintains plasma biomarkers at levels consistent with inhibition of AD pathology and neuroinflammation; PK was not affected by immunogenicity. From modeling data, initiating subcutaneous maintenance dosing of 360 mg weekly at 18‐ or 24‐months results in similar effect on amyloid PET and CDR‐SB compared to biweekly dosing for 4 years. There was no ARIA‐E, ARIA‐H, or deaths reported in participants receiving 360 mg subcutaneous lecanemab. Injection site reactions were infrequent and mostly of mild or moderate severity. Systemic reactions to subcutaneous treatment were uncommon. Among dosing options tested, lecanemab subcutaneous 360 mg autoinjector was selected as the optimal formulation for maintenance therapy.

**Conclusions:**

Subcutaneous lecanemab has low‐risk for immunogenicity. Lecanemab, administered subcutaneously, has a safety, tolerability, and pharmacodynamic profile that favorably compares to the approved 10mg/kg biweekly intravenous dose, and autoinjector administration provides greater patient convenience.

